# Floral phenology of an Andean bellflower and pollination by buff‐tailed sicklebill hummingbird

**DOI:** 10.1002/ece3.8988

**Published:** 2022-06-05

**Authors:** Mannfred M. A. Boehm, David Guevara‐Apaza, Jill E. Jankowski, Quentin C. B. Cronk

**Affiliations:** ^1^ Department of Botany University of British Columbia Vancouver British Columbia Canada; ^2^ Biodiversity Research Centre University of British Columbia Vancouver British Columbia Canada; ^3^ Facultad de Ciencias Biológicas Universidad San Antonio Abad del Cusco Cusco Peru; ^4^ Department of Zoology University of British Columbia Vancouver British Columbia Canada

**Keywords:** Andes, Campanulaceae, *Centropogon*, diversification, *Eutoxeres*, specialization, steady‐state flowering, trapline

## Abstract

The Andean bellflowers comprise an explosive radiation correlated with shifts to specialized pollination. One diverse clade has evolved with extremely curved floral tubes and is predicted to be pollinated exclusively by one of two parapatric species of sicklebill hummingbirds (*Eutoxeres*). In this study, we focused on the floral biology of *Centropogon granulosus*, a bellflower thought to be specialized for pollination by *Eutoxeres condamini*, in a montane cloud forest site in southeastern Peru. Using camera traps and a pollination exclusion experiment, we documented *E*. *condamini* as the sole pollinator of *C*. *granulosus*. Visitation by *E*. *condamini* was necessary for fruit development. Flowering rates were unequivocally linear and conformed to the “steady‐state” phenological type. Over the course of >1800 h of monitoring, we recorded 12 *E*. *condamini* visits totaling 42 s, indicating traplining behavior. As predicted by its curved flowers, *C*. *granulosus* is exclusively pollinated by buff‐tailed sicklebill within our study area. We present evidence for the congruence of phenology and visitation as a driver of specialization in this highly diverse clade of Andean bellflowers.

## Graphical abstract

Specialized pollination is thought to drive niche partitioning in plants and hummingbirds. Floral curvature is one mode by which specialization is thought to operate, but many pollinator species are elusive and understudied. In this study, we document, for the first time, specialized pollination in the rarely seen buff‐tailed sicklebill hummingbird and the Andean bellflower *Centropogon granulosus*. Photograph taken by Gloria Jilahuanco (Asociación para la Conservación del Valle de Kosñipata, APCONK). Photo used with permission.

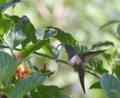

## 1 INTRODUCTION

The Andean bellflowers (Lobelioideae) of *Centropogon*, *Siphocampylus*, and *Burmeistera* (the “centropogonids”) comprise over 550 species emerging in the last five million years (Lagomarsino et al., 2016). This rapid diversification is correlated, in part, with the repeated evolution of pollination by bats and hummingbirds (Lagomarsino et al., 2017). For some centropogonids, pollinator shifts are concomitant with pollinator specialization which can initiate or reinforce reproductive isolation (Lagomarsino & Muchhala, 2019). That is, a pollinator shift may incidentally reduce the number of effective pollinators and in some cases, lower interspecific pollen transfer (Armbruster, 2017). Although it is unknown whether specialization is a cause or consequence of rapid radiations, it is likely to play a role in the maintenance of diversity in this species‐rich clade (Armbruster, 2014; Kay & Sargent, 2009).

One diverse clade of *Centropogon*, the “eucentropogonids” (38 spp.), evolved after a single unique shift to pollination by sicklebill hummingbirds (*Eutoxeres*, Lagomarsino et al., 2017). Almost all members of this clade are predicted, on the basis of their strongly curved corolla tubes, to be pollinated by sicklebills (Stein, 1987). In this study, we focus on the pollination of *Centropogon granulosus* C. Presl by buff‐tailed sicklebill (*Eutoxeres condamini*).

**FIGURE 1:**
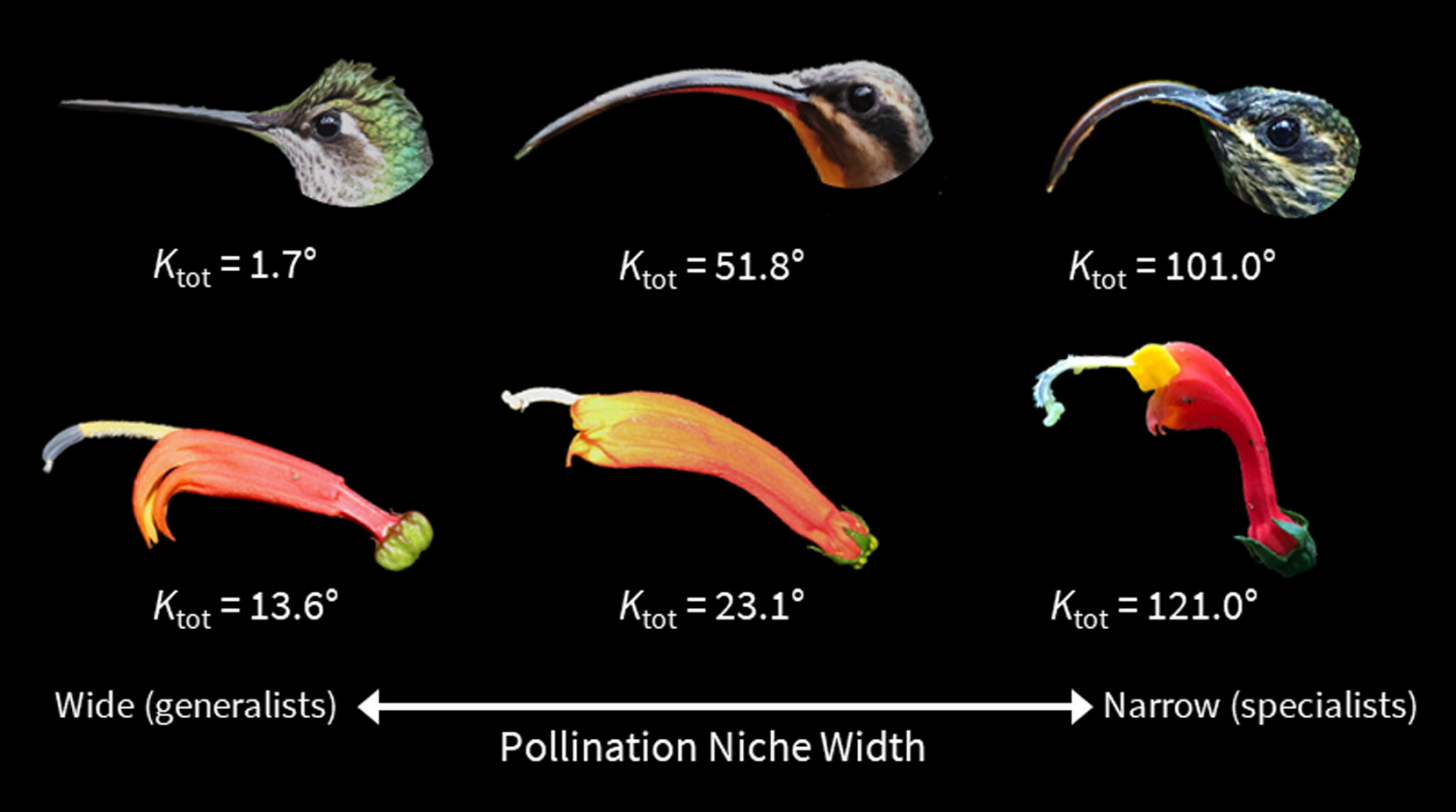
Presumed pollination niches within hummingbird‐adapted Centropogon. $K_{\mathrm{tot}}$ is total curvature in degrees (see: Boehm et al., 2022). Species in reading order: *Eugenes fulgens* (photo: Ronald E. Thill). *Phaethornis guy* (photo: Mike Hooper). *Eutoxeres condamini* (photo: Julian Heavyside). *C*. *valeroi* (photo: Laura Lagomarsino). *C*. *solanifolius* (photo: Terry Gosliner). *Centropogon granulosus* (photo: Josh Vandermeulen). Evidence for pollination in each species pair is described in: Colwell (1973); Snow (1977); this study

*Centropogon granulosus* is an understory vine with abruptly curved, bright red to orange tubular flowers (Figure 1). This species is both the most widespread and variable eucentropogonid, occurring from southern Nicaragua to Bolivia. The species examined here conforms to *Centropogon granulosus* subsp. *granulosus* (sensu Stein, 1987). Although other eucentropogonid species are found in this region (Stein, 1987), we focus on *C*. *granulosus* as it has been previously studied in Costa Rica with respect to pollination by another sicklebill species, *Eutoxeres aquila* (Stiles, 1985), the only congener of *E*. *condamini*. Moreover, *C*. *granulosus* is locally abundant, providing a tractable system for study.

*Eutoxeres* is comprised of two parapatric species of sicklebill hummingbirds that together, adhere to the geographic distribution of the eucentropogonids (Abrahamczyk et al., 2017). White‐tipped sicklebill (*Eutoxeres aquila*) occurs from Costa Rica to northern Peru, whereas buff‐tailed sicklebill (*E*. *condamini*) occurs from northern Peru to Bolivia (Hinkelmann & Boesman, 2020). Previous studies have supported white‐tipped sicklebill as a specialized pollinator of eucentropogonids and some *Heliconia* spp. with curved corolla tubes (Gill, 1987; Maglianesi et al., 2015; Morrison & Mendenhall, 2020; Stiles, 1985). Its bill curvature matches the curved corollas of these plants more than other co‐occurring hermits (Maglianesi et al., 2014; Sonne et al., 2019). Further, its local abundance is correlated with the seasonal flowering of *C*. *granulosus* in Costa Rica (Stiles, 1985). In contrast, very little is known of its southern congener, *E*. *condamini*. Like *E*. *aquila*, its curved bill appears to be suited to feed from eucentropogonids. Currently, there is only a single written record of visitation to a eucentropogonid (*Centropogon gamosepalus* Zahlbr., Stein, 1987), and further details on the extent of mutualism have not yet been studied (e.g., effects of visitation on fruit set and seed production).

Furthermore, because this pollination system is thought to be specialized, we expect additional aspects of the pollination syndrome, specifically phenology, to reflect adaptation to *Eutoxeres* behavior. In addition to the seasonal flowering trends documented by Stiles (1985), phenological patterns at finer temporal scales (i.e., days) might also conform to the daily foraging habits of *Eutoxeres*. Phenological patterns at this scale have been previously categorized by Gentry (1974): for example, “big bang” species produce many flowers simultaneously over several days, whereas “steady‐state” species produce only a few flowers per day over a number of weeks. Considering that *E*. *aquila* is a trapliner (Stiles, 1985; but see Sargent et al., 2021), the “phenological type” of Gentry (1974) that eucentropogonids are likely to exhibit is steady‐state flowering, consistent with low, but regular, daily visitation rates by pollinators. Moreover, we expect steady‐state flowering to provide insufficient daily nectar to territorial hummingbirds so that these plants would, therefore, only be visited by traplining species (Kessler et al., 2020).

Although the phenological types of some centropogonids have been described qualitatively, (e.g., Colwell et al., 1974; Weiss, 1996), the “phenological type” framework of Gentry (1974) considers two continuous variables, flowering duration (*L*) and rate (*r*). We propose that to categorize phenological types, the anthesis rate (*r*) should be examined for linearity, where we expect steady‐state species to exhibit a constant daily flowering rate, whereas “cornucopia” and “big bang” species would flower nonlinearly (Gentry, 1974). To this end, the *average deviation from linearity* metric (Kroll et al., 2000) will be useful in developing a reproducible, quantitative framework for assigning Gentry’s (1974) phenological types (see Methods).

The goal of this study is to test the hypothesis that eucentropogonids are uniquely specialized for pollination by sicklebill hummingbirds, specifically the less well‐known buff‐tailed sicklebill, by examining the floral phenology and pollination of *C. granulosus*. Specifically, we ask: (1) Is buff‐tailed sicklebill a visitor to, and the sole pollinator of *C*. *granulosus*? (2) Does sicklebill visitation affect the reproductive success of *C*. *granulosus*? and (3) Is the phenological type of *C*. *granulosus* consistent with adaptation to the presumed foraging mode of buff‐tailed sicklebill, that is, does *C*. *granulosus* exhibit steady‐state flowering?

## 2 MATERIALS AND METHODS

### 2.1 Field site

**FIGURE 2:**
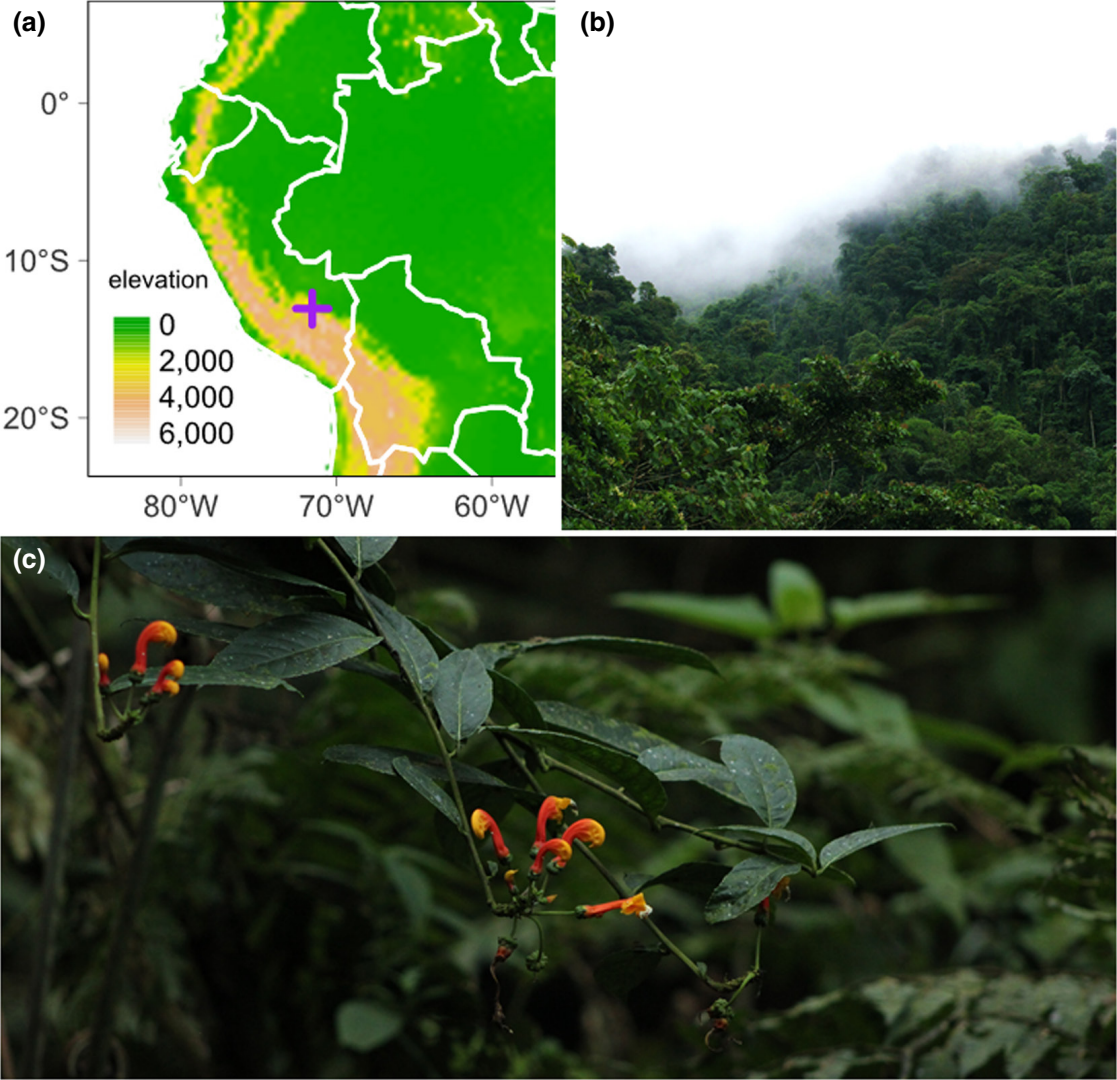
(a) Elevational heatmap of Peru and neighboring countries. Colored cells represent meters above sea level. Map generated using tmap v.3.3‐2 (Tennekes, 2018). The study site location is marked with a cross. (b) Montane cloud forests of the eastern Andes (San Pedro, Peru). (c) *Centropogon granulosus*, an understory vine often flowering at forest edges and treefall gaps

We based our fieldwork at the Cock‐of‐the‐Rock Lodge situated at ~1350 m a.s.l. in the Kosñipata Valley, Department of Cusco, Peru (−13.055, −71.548 DD). Research Permit #0441‐2017 was administered by the Servicio Nacional Forestal y de Fauna Silvestre (SERFOR). The field site is stationed at the transition of lower montane forest and cloud forest within the Yungas ecoregion on the eastern slope of the Peruvian Andes (Figure 2). The local mean annual rainfall and temperature are 2631 mm and 19.1°C, respectively (Salinas et al., 2011).

### 2.2 Pollinator observations

We deployed five camera traps (Hyperfire HC600; Reconyx Inc.) near *Centropogon granulosus* vines located in a previous survey of the area (Boehm et al., 2018). Cameras were mounted onto nearby trees using a bungee cord, typically at a distance of 1–2 m from (and at a height equivalent to) the inflorescence. Camera traps were checked for new captures every 12 h. If no floral visitors were recorded within 3 days, the camera traps were moved to different *C*. *granulosus* individuals. Where floral visitors were recorded, we attempted targeted video recording to better document visitation behavior. Camera traps were active continuously from August 17 to September 20, 2017 (Table S1).

### 2.3 Pollinator exclusion and floral development

Following the methods of Sun et al. (2017), we constructed and deployed wire cages covering one inflorescence each from six *Centropogon* individuals (Figure S3). Wire cages prevent hummingbirds from accessing the flowers while allowing invertebrates to move freely. An additional 10 inflorescences (one per individual plant) were marked with cardstock tags tied to the stem and monitored as controls. Using the control flowers, we defined eight stages of floral development (A–H; Table S2, Figure 4). These stages were used to quantify and compare the developmental trajectories of the control and pollinator‐excluded flowers. Monitoring of a flower stopped when (1) berry development completed (stage H), (2) the flower died prematurely, or (3) the study period ended. Daily observations were recorded between August 17 and September 20, 2017. Floral development data were analyzed in R v.4.0.2 (R Core Team, 2017).

Some flowering stages were not completely observed due to herbivory or weather. Similarly, monitoring of some flowers began with the current stage partially completed. This type of data is “right censored”, that is, the true durations of these stages are greater than was observed (Allison, 2014). To account for censoring, we fit parametric survival functions (Allison, 2014) to the stage duration data. This allowed an estimation of the median duration ($m_{X_{n}}$) for each stage (*X_n_*), that is, the number of days elapsed in stage *X_n_* before the daily probability of transitioning to stage *X_n_*_+1_ surpassed 50%. Survival functions and median stage durations were estimated from the Gompertz distribution (Ricklefs & Scheuerlein, 2002) using flexsurv v.2.0 (Jackson, 2016).

To reconstruct floral development from the censored dataset, we used the median stage durations and 95% confidence intervals (CI) estimated from the survival analysis above. For each treatment, we cumulatively summed the median stage durations to approximate the number of days elapsed between stages A and G. We accounted for error propagation, that is, the uncertainty of each $m_{X_{1}},\cdots ,m_{X_{n}}$ in influencing the 95% CI of $m_{X_{1}}+\cdots +m_{X_{n}}+m_{X_{n+1}}$, by summing the 95% CIs in quadrature (Ku, 1966).

### 2.4 Phenological type

To characterize the phenological type of *C*. *granulosus*, we used broom v.0.7.6 (Robinson et al., 2021) to fit linear models to the number of flowers produced through time. A separate model was fit for each inflorescence that produced at least five flowers (*n* = 5 for each treatment). The slope of each linear regression was interpreted as the anthesis rate. To assess linearity, we used lin.eval v.0.1.2 (Shrivastav, 2019) to fit linear and polynomial (>1°) curves to the anthesis rate. This method uses the *average deviation from linearity* (Kroll et al., 2000), to determine if non‐linear fits have significantly lower residuals than a linear regression.

## 3 RESULTS

### 3.1 Floral visitors

**FIGURE 3:**
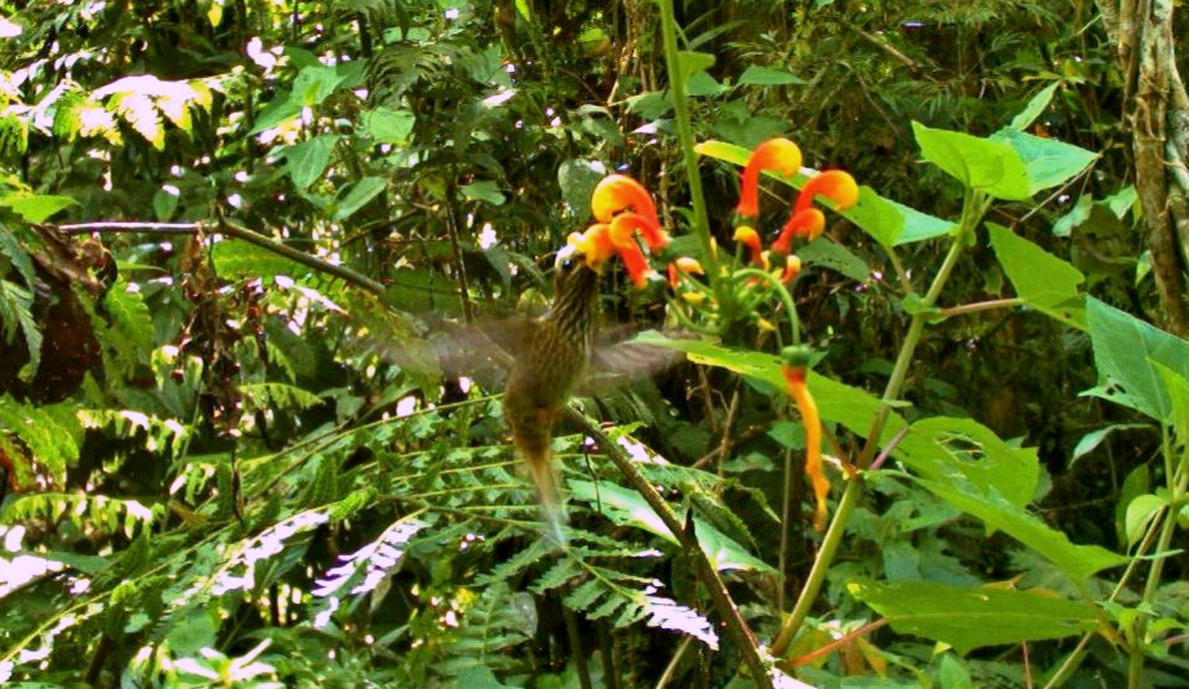
Visitation of *Centropogon granulosus* by Buff‐tailed Sicklebill (*Eutoxeres condamini*)

Camera trap recordings and *in situ* observations confirm buff‐tailed sicklebill (*E*. *condamini*) as a visitor to *Centropogon granulosus* flowers (Figures 3, S4). Visitation tended to occur from 5:20 to 10:40 in the morning (*n* = 9), and 12:40 to 16:30 in the afternoon (*n* = 3), though these patterns may have been affected by our activity in the area. Given that *Eutoxeres* is active within an ~11‐h daily window, the total monitoring effort was ~1870 h (5 camera traps × 34 days × 11 h per day). Within this time, we recorded 12 visits to six *C*. *granulosus* individuals, totaling 42 s of *E*. *condamini* observation (Figure S12, Table S3). Ten of the 12 records were single, brief visits (≤3 s) that occurred once in the day—two additional records were made when a second visitation was observed on the same day. A total of seven flowers were probed from six *C*. *granulosus* individuals, that is, a second visit was recorded to an inflorescence as flowers opened sequentially. *E. condamini* feeds both by perching on the lignified inflorescence (*n* = 3) and hovering (*n* = 9). We also recorded two instances of sicklebills approaching and inspecting inflorescences without open flowers. Wedge‐billed hummingbird (*Schistes geoffroyi*) was also recorded nectar robbing *C*. *granulosus* by piercing the corolla tube at the base. Over the course of 2 days, a camera trap recorded five visits per day to the same inflorescence (Table S4). Further details of *S*. *geoffroyi* behavior can be found in Boehm (2018). No other hummingbirds were recorded visiting these flowers.

Reviewing still frames from the video recording reveals an interesting feeding problem posed by the sharply curved flowers of *C*. *granulosus*. The hook shape forces the hummingbird to hover below the corolla opening and tilt its head backward so that it is facing skyward (dorsal head flexion), at which point, it can insert the tip of its bill into the flower aperture (Figure S4). The remainder of the bill is further inserted by tilting the head back to a forward‐facing position while lifting itself to eye level with the corolla opening. Due to the unique morphology and orientation of *C*. *granulosus* flowers, this hovering maneuver is likely performed only by *Eutoxeres*. We note that once the bill is inserted, the throat and crown are covered by the ventral and dorsal corolla lobes, respectively.

Two additional vertebrates, a murid (Muridae) and a long‐nosed bat (Glossophaginae) were recorded near the inflorescences but not observed to interact with the plant directly (Figures S5, S6). We note this because it is unknown how the fleshy berries are dispersed, though we documented signs of frugivory (Figure S7).

Numerous invertebrates occupied or visited the flowers of *C*. *granulosus* in this study. As found in previous studies, we observed ants (Stein, 1992), mites (Naskrecki & Colwell, 1998), and dipterids (Weiss, 1996) in or on the flowers of this species. We observed unidentified Arachnids inside of the floral tubes, and note that *Anelosimus* spiders (Araneae) are known to build webs scaffolded by *Centropogon coccineus* (Hook.) Regel ex B.D. Jacks. (Nentwig & Christenson, 1986). We also recorded a larval lepidopteran inhabiting a flower (Figure S8), and a stingless bee (Meliponini) collecting pollen from the anther scale (Figure S9).

### 3.2 Pollinator exclusion and floral development

**FIGURE 4:**
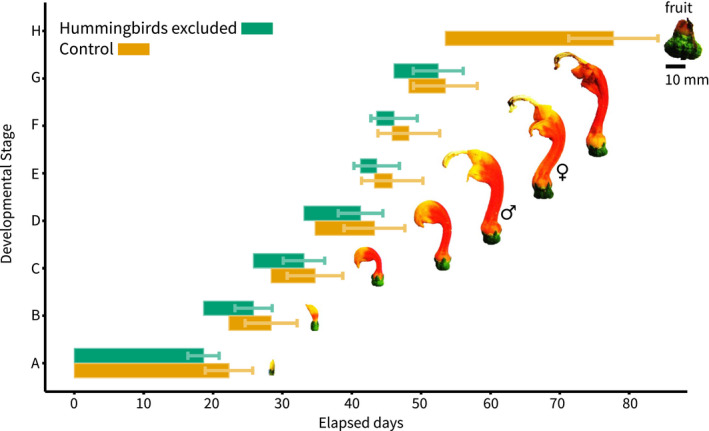
Developmental trajectories for the flowers of *Centropogon granulosus* and effects of pollinator exclusion (green) versus the control treatment (orange). The bars represent the median duration spent in each stage. 95% of CIs are estimates of when a stage could end. At stage E, where some individuals begin, others will have already finished and moved on to stage F. No fruits were produced by plants with pollinators excluded

We identified and described eight stages of floral development in *C*. *granulosus* (Table S2, Figure 4). During the first stage (A), the flowers are small buds and have not yet developed curvature. In stages B to D, the flowers elongate and form their characteristic hook shape—nearly all floral curvature is developed here. Stages E and F are defined by the staminate and pistillate phases of anthesis, respectively. Following anthesis, the flowers wilt (Stage G) and produce berries (Stage H, Table S2).

These eight stages were used to compare developmental differences between control and pollinator‐excluded flowers (Figure 4). Between treatments, the progression of floral development is comparable from stages A (bud development) to E (anthesis). However, control flowers spend 24.2 ± 4.47 days (median ± 95% CI) developing berries, while no hummingbird‐excluded flowers produced berries.

### 3.3 Flowering rate

Hummingbird exclusion did not affect the total number of flowers produced (*p* = .782, *t*_14_ = 0.282, *d* = 0.15). The caged inflorescences produced 11.2 ± 4.6 flowers over the study period (34 days), whereas controls produced 12.2 ± 8.1 flowers (mean ± SE). The upper limit of flower production for a single inflorescence has not been determined, though we counted 68 flower abscission scars on the peduncle of an individual not included in this study (Figure S10).

**FIGURE 5:**
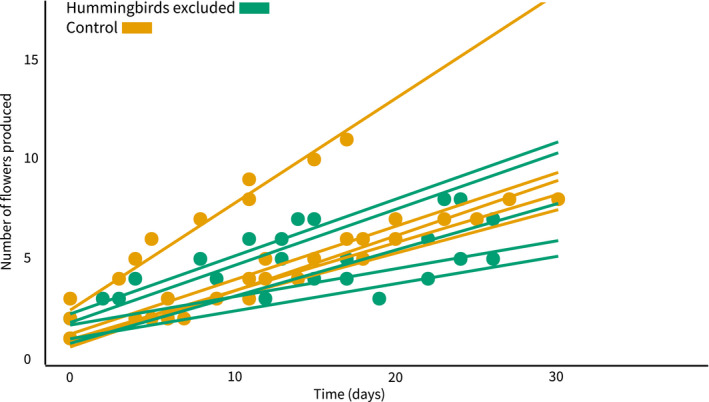
Flowering rates in *Centropogon granulosus*. The *x*‐axis represents the number of days elapsed since the first flower opened. The *y*‐axis counts the cumulative number of flowers opened since *t* = 0, not the number of flowers open simultaneously. Each line shows the flowering rate of an individual belonging to the exclusion treatment (green) or control group (orange). Rates vary between individuals (*p* = .027, *t*_8_ = 2.70, *d* = 1.91), but do not vary between treatments (*p* = .200, *t*_8_ = 1.40, *d* = 0.99)

Linear models accurately described flowering rate (Figure 5): all anthesis rates were fit better by linear models than polynomials (*p* < .05). Flowering rate (slope) varied among inflorescences (*p* = .027, *t*_8_ = 2.70, *d* = 1.91) but not between treatments (*p* = .200, *t*_8_ = 1.40, *d* = 0.99). The average rate was one anthesis event per 3.23 ± 0.12 days for control inflorescences and 4.60 ± 0.07 days^−1^ for pollinator‐excluded inflorescences. The highest flowering rate was documented in a control plant at 1.88 days^−1^, nearly twice the rate of the next fastest individual (Figure 5). When the fast inflorescence is removed, the mean flowering rate for control plants is 3.94 ± 0.03 days^−1^. The lowest flowering rates were 7.22 days^−1^ and 7.06 days^−1^, both in pollinator‐excluded plants.

## 4 DISCUSSION

### 4.1 Buff‐tailed sicklebill is a pollinator of *C. granulosus*

As predicted from its extreme bill curvature, buff‐tailed sicklebill (*E*. *condamini*) is a visitor to *C*. *granulosus*, and these visits are necessary for developing fruit. No other hummingbirds were observed legitimately probing these flowers. Covering flowers with wire cages excluded hummingbirds while allowing invertebrates to access the flowers freely—however, none of these flowers produced fruits. Therefore, we conclude that buff‐tailed sicklebill is the sole pollinator of *C*. *granulosus*.

While probing for nectar, the face of *E*. *condamini* is inserted into the corolla tube so that the crown and throat are covered by the petal lobes. This is facilitated by the exceptionally inflated corolla opening characteristic of the eucentropogonids (Lagomarsino et al., 2017). While narrow corolla apertures are thought to promote specialization (Temeles et al., 2002), the evolution of curvature might relax selection for corolla width. Conversely, because *E*. *condamini* tilts its head backward during bill insertion, it may not be able to see the corolla opening; thus, a narrow corolla width could negatively affect pollination if the barriers to accessing nectar are too high (Rico‐Guevara et al., 2021; Westerkamp, 1990).

In contrast to previous accounts of sicklebill visitation to *Centropogon* (Stein, 1987; Stiles, 1985), we observed hovering in addition to perching. While floral orientation in some hummingbird‐pollinated plants may have evolved to exclude non‐hovering visitors (R. Colwell, pers. comm.), hovering is one of the most energetically expensive modes of locomotion (Suarez & Gass, 2002) and is avoided when perches are available (Westerkamp, 1990). Recent work has found that short‐billed hummingbird species have repeatedly evolved large claws that improve their ability to perch (R. Colwell, pers. comm.). Conversely, long‐billed species tend to hover to feed, supporting the idea that long (and sometimes curved) tubular flowers evolve in response to selection for pollinator specialization (Temeles et al., 2019). We speculate that the inflorescences of *C*. *granulosus* are lignified primarily to support and orient flowers and are only opportunistically used by sicklebills as perches. This is because open flowers tend to face away from the stem on long pedicels (Figure 2). This is in contrast to *E*. *condamini* visits to nearby *Heliconia*, which has flowers oriented so that the aperture is aligned with the perch (i.e., floral bract, Figure S11). Whether floral orientation promotes specialization in the eucentropogonids is an understudied aspect of pollination in this clade.

### 4.2 Steady‐state flowering and traplining

Because hummingbird species generally adhere to a single foraging mode (Feinsinger & Colwell, 1978; Stiles, 1985; but see Sargent et al., 2021), phenological types may be effective filters of the local pollinator community, further promoting floral specialization in the eucentropogonids. As with floral shape, phenological types are thought to evolve either via competition for pollination or selection against interspecific pollen transfer (Kessler et al., 2020; Primack, 1985; Rathcke & Lacey, 1985). Therefore, accurately assigning phenological types in the context of pollinator foraging modes will be a key to examining the evolution of this trait in the centropogonids and assessing the role of phenology in pollinator shifts.

*Centropogon granulosus* exhibits a linear flowering rate befitting the “steady‐state” phenological type described by Gentry (1974) as “[the production of] a few flowers a day over an extended period of time (usually a month or more)”. It is one of several phenological modes representing an axis of niche partitioning that is thought to contribute to tropical plant diversity (Gentry, 1974; Kessler et al., 2020). Indeed, most hummingbird species exhibit foraging behavior that is adapted either to steady‐state or “cornucopia” flowering (sensu Gentry, 1974), with few species able or willing to visit plants of both types (Kessler et al., 2020). However, beyond qualitative descriptors, there is a need for a quantitative framework to better define and classify phenological types. Because the steady‐state strategy implies a linear flowering rate, anecdotal observations of phenological type can be tested using the linearity metric implemented here.

Despite the continental breadth of the *C*. *granulosus* complex, there is a striking similarity in seasonal flowering duration across its range. Stiles (1985) recorded a 9‐month (~270 days) flowering season of *C*. *granlosus* in Costa Rica. Considering the peduncle with 68 pedicel scars (see: Results), and the mean flowering rate of 3.94 ± 0.03 days (controls), we estimate that this inflorescence produced flowers for 268 ± 2.04 days. Not only is this a remarkably long flowering season for one individual inflorescence, but this phenological type might occur across the range of *C*. *granulosus* pollinated by either species of *Eutoxeres*.

Sicklebills were not marked and our ability to comment on individual behavior is limited. Nonetheless, the visitation rates support the notion that buff‐tailed sicklebill is a trapliner. More specifically, this species appears to exhibit “traveling exploitation” (sensu Sargent et al., 2021). We make this designation based on the observations that (1) these hummingbirds have not been recorded defending static territories; and (2) individual food plants are visited 1–2 times per day for brief periods (seconds) of foraging. This is perhaps the fitness advantage promoting the evolution of specialized pollination in *C*. *granulosus*: Sicklebill visits are infrequent but highly effective in transferring intraspecific pollen, as suggested by the pollinator exclusion experiment. While the behaviors exhibited by *E*. *condamini* are in accordance with the those documented for white‐tipped sicklebill in Costa Rica (Stiles, 1985), the fine‐scale daily movements of *Eutoxeres* (and Hermits generally) have not yet been studied—at present, comparative analyses are constrained by our limited knowledge of these rarely seen pollinators.

Finally, while steady‐state flowering is not solely indicative of specialization in *Eutoxeres*, we speculate that it is a component of the iterative process by which specialization evolves. That is, steady‐state flowering may have first co‐evolved with traplining hummingbirds (Rombaut et al., 2022), which excluded visitation by species under stabilizing selection for territoriality. Among the steady‐state species, floral morphology continued to evolve, further partitioning the steady‐state species between grades of curvature (Figure 1).

## 5 CONCLUSION

Pollinator specialization is likely to play an important role in the generation and/or maintenance of species in the mega‐diverse Andean Lobelioids. In this study, we confirmed the prediction that *C*. *granulosus* is pollinated exclusively by buff‐tailed sicklebill (*E*. *condamini*) within our study site, where its congener, white‐tipped sicklebill (*E*. *aquila*), is absent. Furthermore, because sicklebills exhibit traplining, we find evidence that specialization operates not only through corolla shape, but also the steady‐state flowering strategy. By documenting plant‐pollinator interactions and phenological type, we hope to provide valuable ecological and natural history data needed to test the role of specialization in the rapid diversification of the Andean bellflowers.

## AUTHOR CONTRIBUTIONS

**Mannfred M. A. Boehm:** Conceptualization (lead); Data curation (lead); Formal analysis (lead); Funding acquisition (equal); Investigation (lead); Methodology (equal); Project administration (equal); Validation (equal); Writing – original draft (lead); Writing  – review & editing (equal). **David Guevara‐Apaza:** Investigation (supporting); Project administration (equal); Resources (supporting); Writing – review & editing (supporting). **Jill E. Jankowski:** Conceptualization (supporting); Funding acquisition (equal); Investigation (supporting); Project administration (supporting); Resources (lead); Supervision (lead); Writing – review & editing (equal). **Quentin C. B. Cronk:** Conceptualization (equal); Funding acquisition (equal); Project administration (supporting); Resources (lead); Supervision (lead); Validation (equal); Writing – review & editing (equal).

## CONFLICT OF INTEREST

The authors declare no conflict of interest.

## Supporting information

Figure S1

Figure S2

Figure S3

Figure S4

Figure S5

Figure S6

Figure S7

Figure S8

Figure S9

Figure S10

Figure S11

Figure S12

Appendix S1

## Data Availability

All data and R scripts are available in the Dryad repository https://doi.org/10.5061/dryad.ns1rn8pwb. These materials are also available as an RStudio Project at: https://github.com/mannfred/centropogon_eutoxeres.
