# Supplementary figures and images for: Floral phenology of an Andean bellflower and pollination by buff‐tailed sicklebill hummingbird
Source: Ecol Evol. 2022 Jun 5;12(6):e8988. doi: 10.1002/ece3.8988 (PMC9168340; doi:10.1002/ece3.8988)

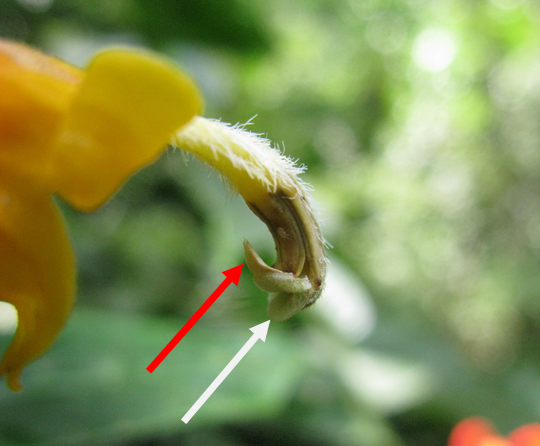

Supplement: Supplementary file 1 — Figure S1 [file ECE3-12-e8988-s007.tif]

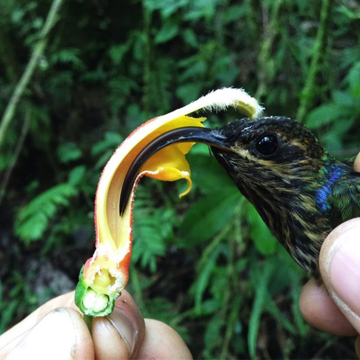

Supplement: Supplementary file 2 — Figure S2 [file ECE3-12-e8988-s006.tif]

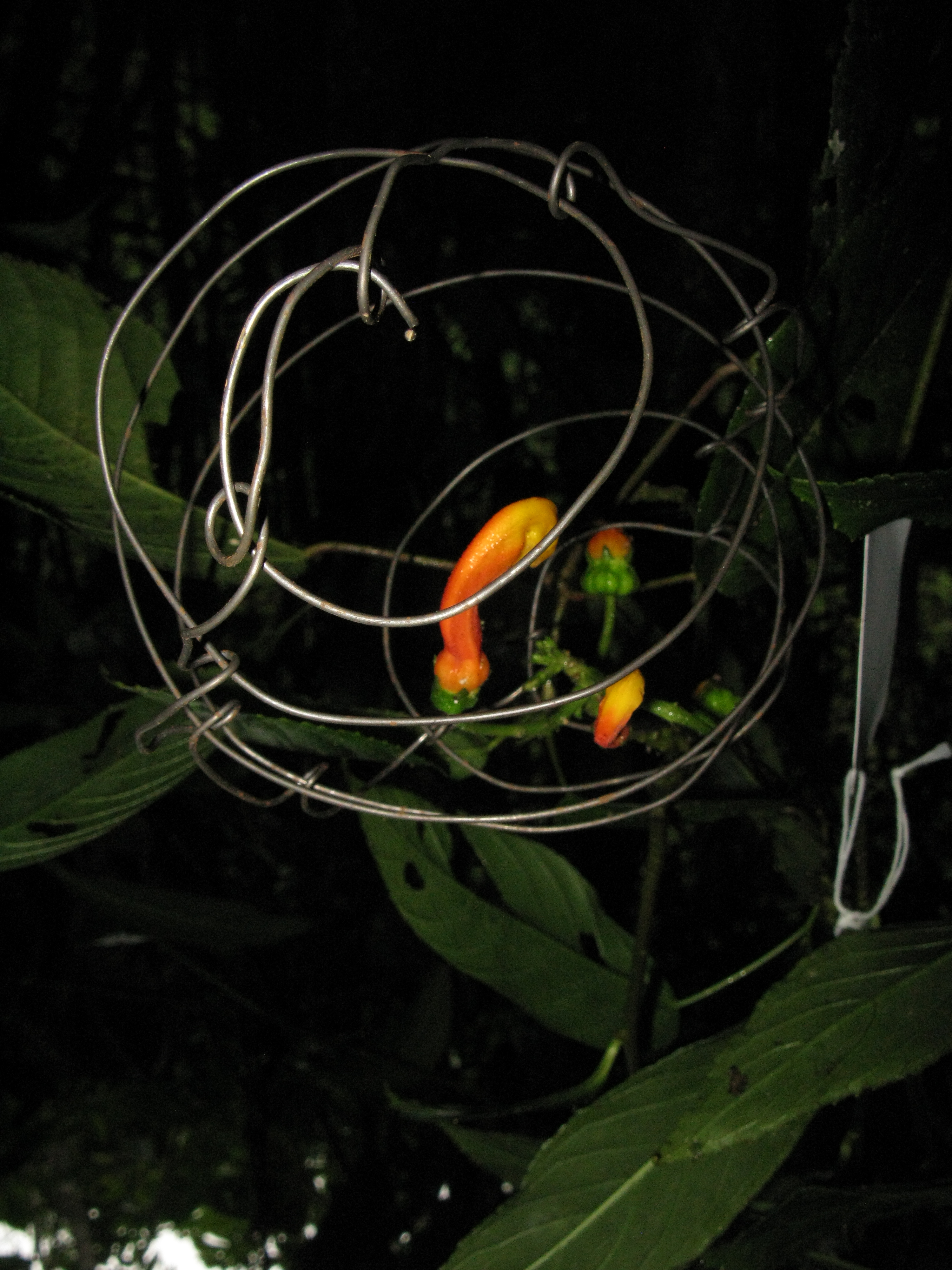

Supplement: Supplementary file 3 — Figure S3 [file ECE3-12-e8988-s001.tif]

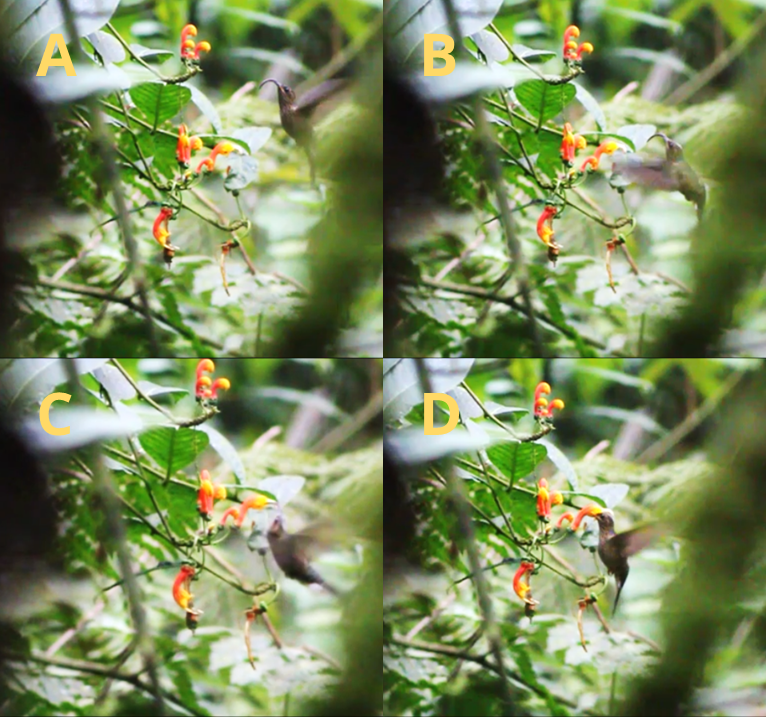

Supplement: Supplementary file 4 — Figure S4 [file ECE3-12-e8988-s011.tif]

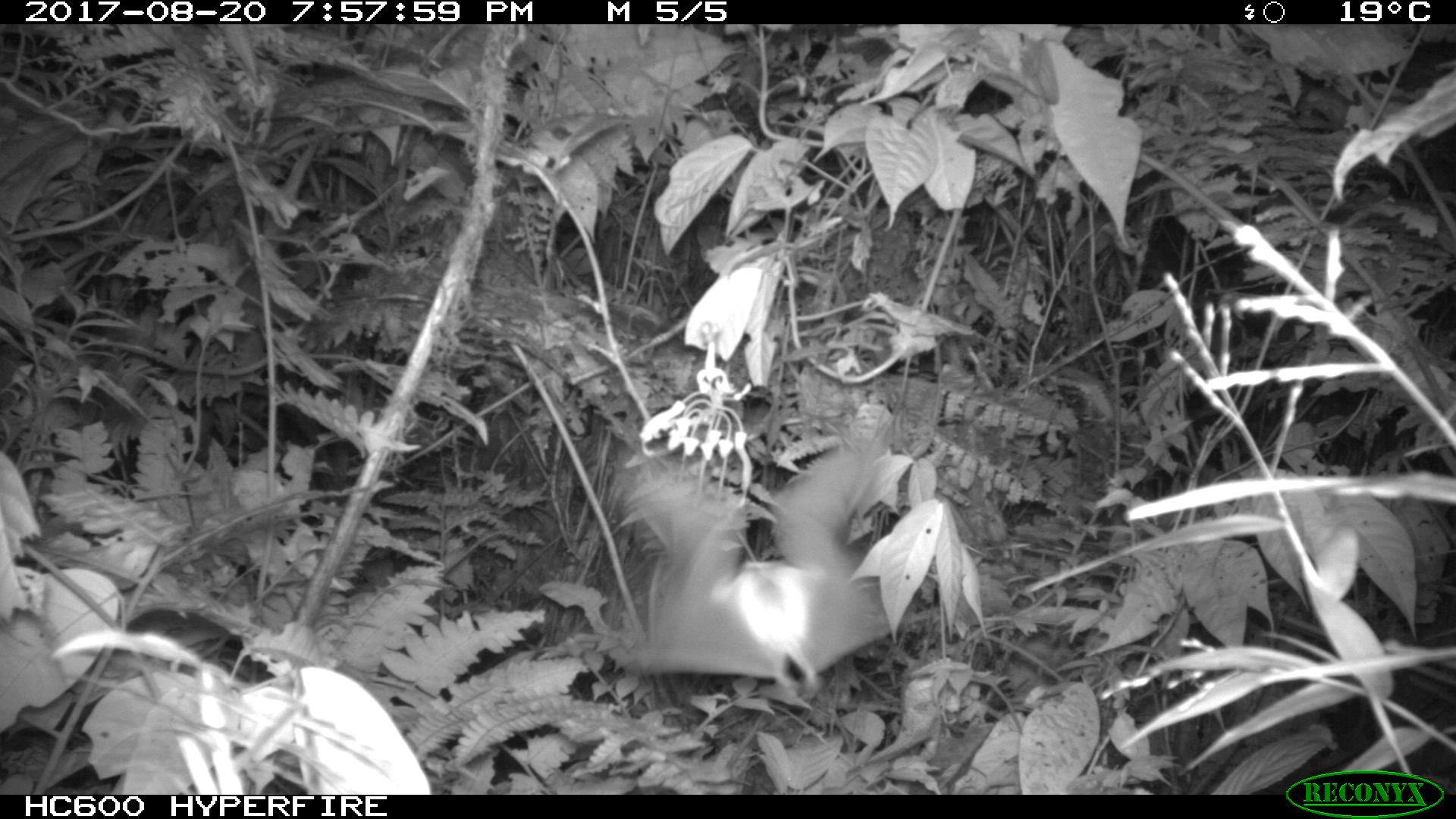

Supplement: Supplementary file 5 — Figure S5 [file ECE3-12-e8988-s008.tif]

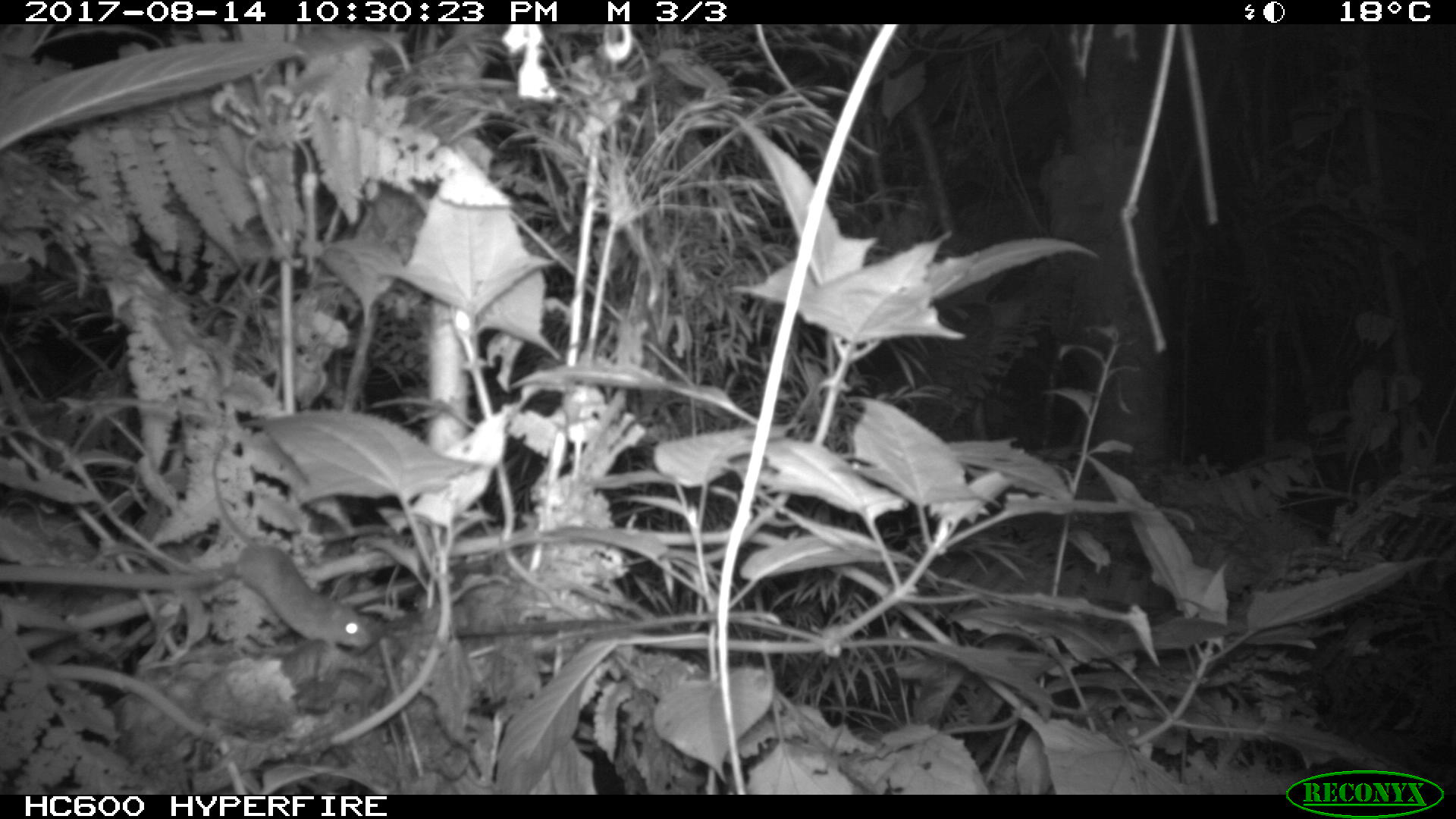

Supplement: Supplementary file 6 — Figure S6 [file ECE3-12-e8988-s003.tif]

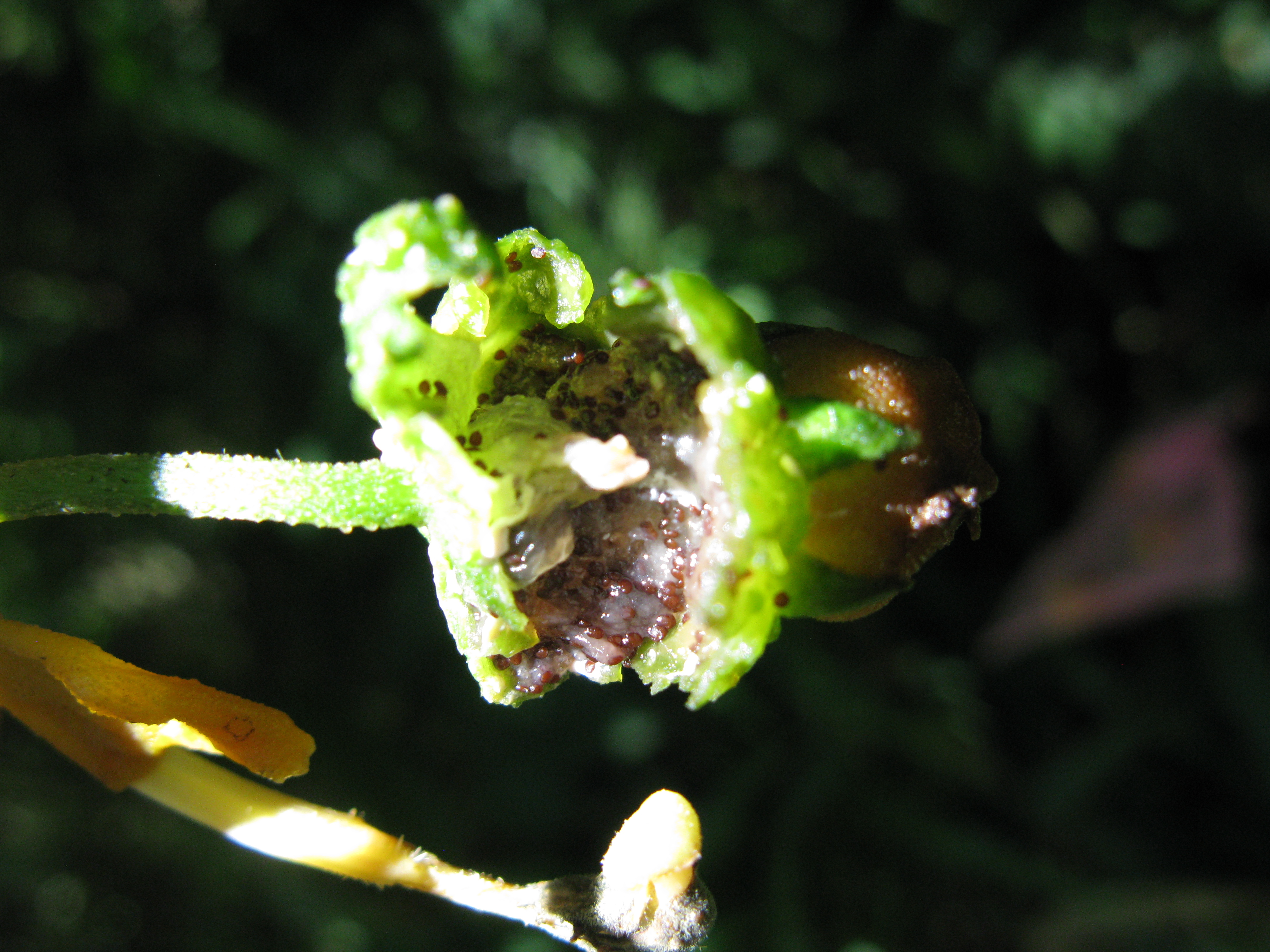

Supplement: Supplementary file 7 — Figure S7 [file ECE3-12-e8988-s012.tif]

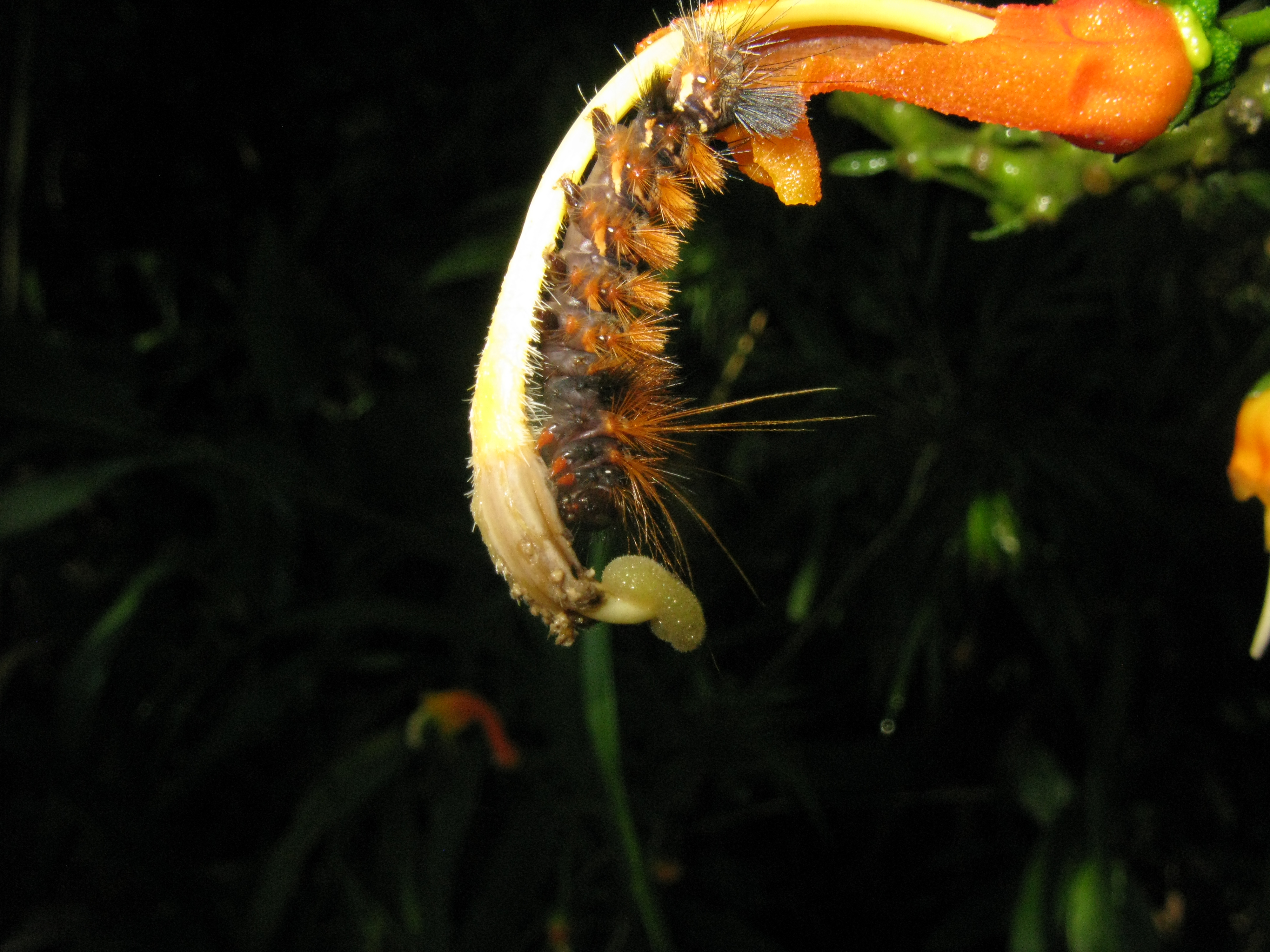

Supplement: Supplementary file 8 — Figure S8 [file ECE3-12-e8988-s002.tif]

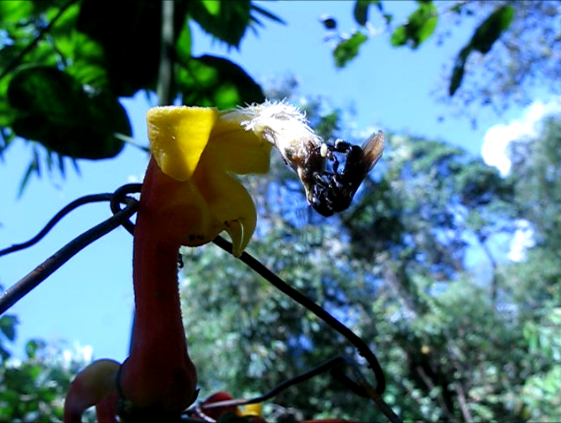

Supplement: Supplementary file 9 — Figure S9 [file ECE3-12-e8988-s013.tif]

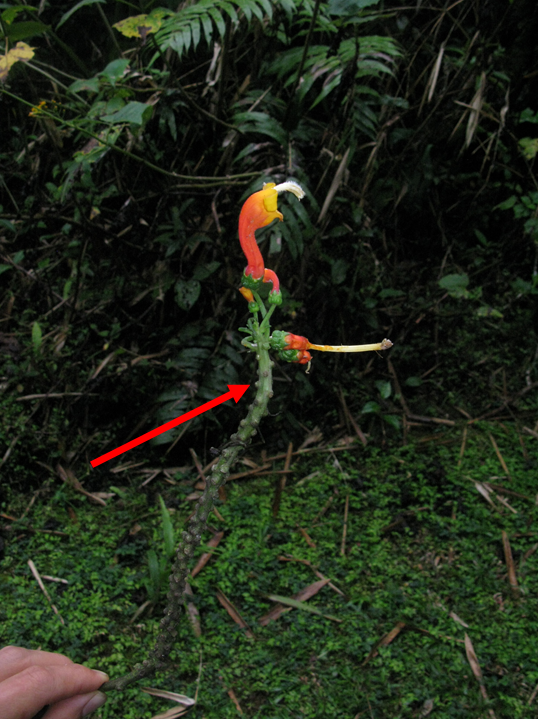

Supplement: Supplementary file 10 — Figure S10 [file ECE3-12-e8988-s009.tif]

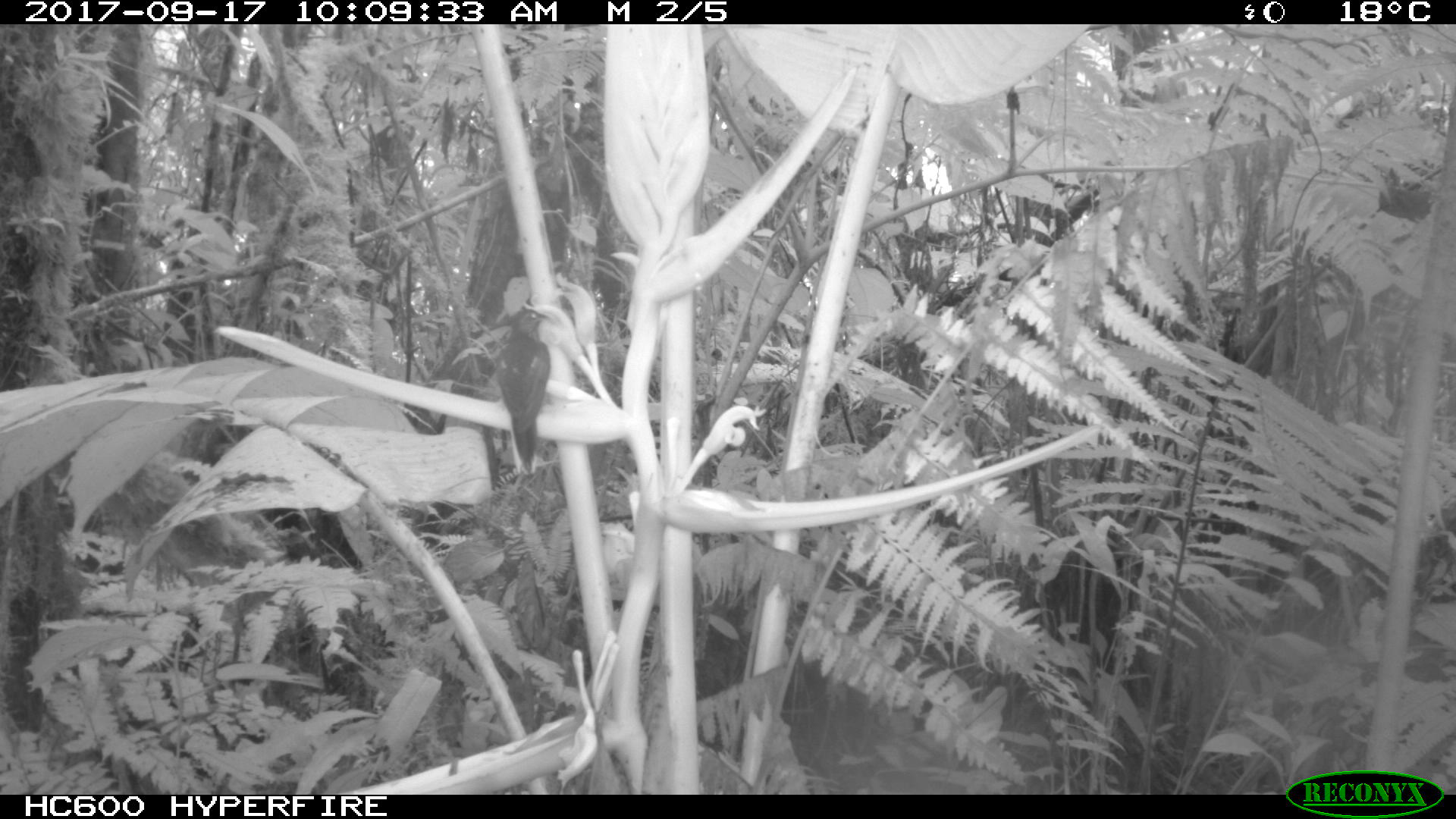

Supplement: Supplementary file 11 — Figure S11 [file ECE3-12-e8988-s010.tif]

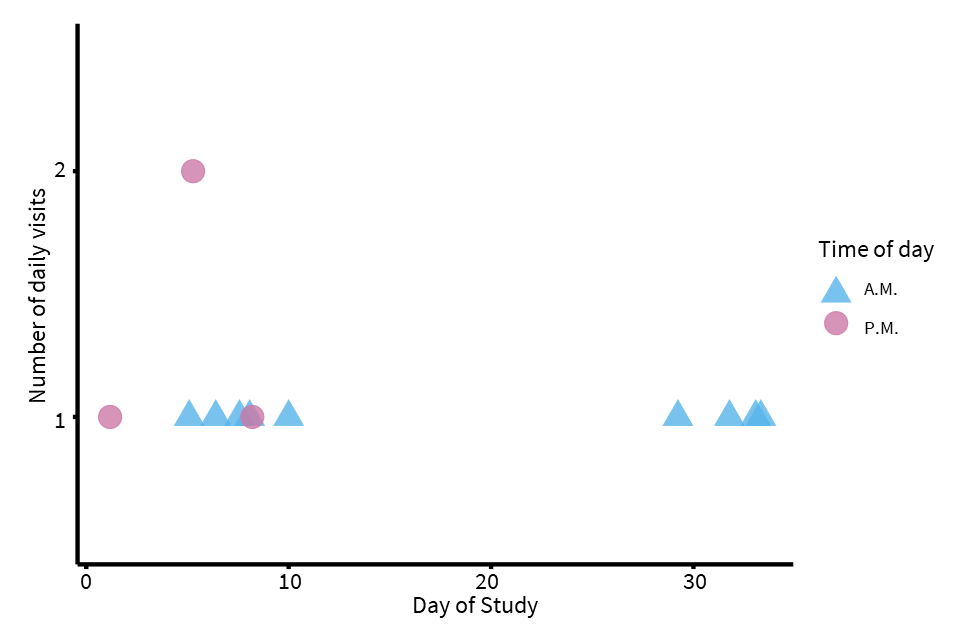

Supplement: Supplementary file 12 — Figure S12 [file ECE3-12-e8988-s005.tif]
