# Supplementary material for: Floral phenology of an Andean bellflower and pollination by buff‐tailed sicklebill hummingbird
Source: Ecol Evol. 2022 Jun 5;12(6):e8988. doi: 10.1002/ece3.8988 (PMC9168340; doi:10.1002/ece3.8988)
Supplement: Supplementary file 13 — Appendix S1 [file ECE3-12-e8988-s004.docx]

**Appendix for: Floral phenology of an Andean bellflower and pollination by Buff-tailed Sicklebill**

#### Further notes on methods

#### *Visit duration*

To estimate the duration of *E. condamini* visits, we considered that the camera traps took photos in sets of five, lasting less than 3 seconds. To make a conservative estimate, we assume the five photos take 3 seconds. For any 5-photo set in which *E condamini* appears, we assume 3 seconds visitation. For example, during visitation, if *E. condamini* appears in two 5-photo sets, we assume a 2 second (= 6 second) visit, even if the hummingbird appeared in only a subset of the 10 photos.

#### *Landmarking and calculating curvature*

In Figure 1 (main text) we present a graphical illustration of the range of pollination niches within the centropogonids. To compute total curvature (sensu [Boehm *et al.* 2022](#ref-boehm_2022)), we used the following protocol. First, images were imported into *tpsUtil* ([Rohlf 2015](#ref-rohlf_2015)). This .tps file is used by *tpsDig* ([Rohlf 2015](#ref-rohlf_2015)) for landmark assignment. We then placed the following two landmarks on each flower: (A) the base of the dorsal side of the corolla tube where the petals attach to the receptacle and (B) the apex of the dorsal petal. Nine additional sliding semi-landmarks were then placed between the two landmarks, outlining the dorsal arc of the corolla tube. For the hummingbirds, the following two landmarks were placed on each bill: (C) the base of the dorsal side of the upper mandible (exposed), and (D) the apex of the bill. Nine semi-landmarks were placed between these landmarks.

The .tps file generated by *tpsDig* was then imported into R v.4.1.1 via readmulti.tps() from geomorph v.4.0.0 ([Adams & Otárola-Castillo 2013](#ref-adams_2013)). We then fit interpolating splines to each landmark configuration, and computed total curvature using curvr v.0.0.1 ([Boehm 2021](#ref-boehm_2021_b)).

#### *Phenological modeling*

To model flowering phenology of *C. granulosus*, we fit the following linear model to the rate of anthesis and senescence for each inflorescence that produced at least five flowers ($n$ = 5 controls, $n$ = 5 pollinator excluded):

$$n_{i}=\beta_{0}+\beta_{1}*days+\epsilon$$

   Where $n_{i}$ is the cumulative number of flowers produced on an inflorescence at $days$=$i$, $\beta_{0}$ is the intercept, $\beta_{1}$ is the flowering rate, and $\epsilon$ is the residual error.

   To determine if pollinator exclusion affected the total number of flowers produced, we fit the linear model:

$$n_{total}=\beta_{0}+\beta_{1}*treatment+\epsilon$$

   Where $\beta_{0}$ is the intercept, $\beta_{1}$ is a coefficient, and $\epsilon$ is the residual error.

# Appendix Tables

Table A1: Locations in San Pedro monitored for Sicklebill visitation to C. granulosus. The first column lists the C. granulosus individual identifiers.

| name | latitude | longitude | elevation |
| --- | --- | --- | --- |
| CNTRPGN 1-10 | -13.05684 | -71.54469 | 1306.706 |
| CNTRPGN 11 | -13.05603 | -71.54515 | 1301.995 |
| CNTRPGN 12-15 | -13.05928 | -71.54773 | 1381.661 |
| CNTRPGN 16-20 | -13.05900 | -71.54666 | 1323.870 |
| CNTRPGN 21-23 | -13.05921 | -71.54690 | 1330.937 |
| CNTRPGN 24-32 | -13.05932 | -71.54778 | 1345.147 |
| CNTRPGN 33-41 | -13.05821 | -71.54805 | 1359.792 |
| CNTRPGN 42 | -13.05720 | -71.54699 | 1370.250 |
| CNTRPGN 43-50 | -13.05728 | -71.54672 | 1368.063 |
| CNTRPGN 51-53 | -13.05563 | -71.54676 | 1358.099 |
| CNTRPGN 54-60 | -13.05696 | -71.54784 | 1398.831 |

Table A2: Stages of floral development in C. granulosus.

| Stage | Description | Median duration days |
| --- | --- | --- |
| A | Flower primordia appears above bracts. No curvature and red-orange pigmentation is not continuous around the base. Flower up to 9 mm tall (as measured from the top of the bracts). Basal diameter up to 4 mm. | 22.3 |
| B | Initiation of curvature, creating 90 degree angle. Red-orange pigmentation is continuous around the base. Flower up to 10 mm tall. Basal diameter up to 6 mm. | 6.1 |
| C | Growth phase. 180 degree angle formed. Red-orange pigmentation outweighs yellow. Flower 14 to 20 mm tall. Basal diameter 6 - 8 mm. | 6.3 |
| D | Pre-anthesis. >180 degree angle formed. Flower 30 mm tall. Basal diameter 7 - 8 mm. | 8.6 |
| E | Anthesis. Mature male-phase flower. Flower 34 -36 mm tall. Basal diameter 7 - 8 mm. | 2.6 |
| F | Female-phase flower. Pistil grows overtop of the pollen trap. Flower up to 36 mm tall. Basal diameter up to 10 mm. | 2.4 |
| G | Senescing flower. Petals wilting but retained. Basal diameter 8 - 10 mm. | 5.3 |
| H | Berry development. Petals senesced and lost. Basal diameter grows from 11 - 17 mm. | 24.2 |

Table A3: Camera trap records of E. condamini.

| Date observed | Time of day | Feeding mode | Visit duration seconds | Temperature celcius | Centropogon ID | Flower ID |
| --- | --- | --- | --- | --- | --- | --- |
| Aug_18_2017 | 12:36 | hovering | 3 | 19 | 1 | 1 |
| Aug_22_2017 | 10:38 | hovering | 3 | 21 | 1 | 1 |
| Aug_22_2017 | 10:38 | hovering | 3 | 21 | 1 | 2 |
| Aug_22_2017 | 15:54 | hovering | 3 | 20 | 1 | 1 |
| Aug_22_2017 | 10:39 | hovering | 3 | 20 | 2 | 1 |
| Aug_23_2017 | 10:24 | hovering | 3 | 19 | 1 | 2 |
| Aug_24_2017 | 7:17 | perching | 3 | 17 | 1 | 1 |
| Aug_24_2017 | 7:17 | hovering | 3 | 17 | 1 | 2 |
| Aug_25_2017 | 16:27 | inspecting | 3 | 19 | 3 | NA |
| Aug_26_2017 | 5:52 | hovering | 3 | 15 | 4 | 1 |
| Sep_15_2017 | 5:24 | hovering | 3 | 16 | 5 | 1 |
| Sep_16_2017 | 5:56 | inspecting | 3 | 15 | 5 | NA |
| Sep_19_2017 | 5:36 | perching | 3 | 14 | 5 | 2 |
| Sep_19_2017 | 7:15 | perching | 3 | 16 | 6 | 1 |

Table A4: Camera trap records of S. geofryii.

| Date observed | Time of day | Feeding mode | Temperature celcius | Centropogon ID | Flower ID |
| --- | --- | --- | --- | --- | --- |
| Sep_11_2017 | 9:58 | hovering | 20 | 7 | 1 |
| Sep_11_2017 | 13:03 | hovering | 22 | 7 | 1 |
| Sep_11_2017 | 13:55 | hovering | 23 | 7 | 1 |
| Sep_11_2017 | 15:01 | hovering | 23 | 7 | 1 |
| Sep_11_2017 | 16:11 | hovering | 22 | 7 | 1 |
| Sep_14_2017 | 10:44 | hovering | 21 | 7 | 2 |
| Sep_14_2017 | 11:42 | hovering | 22 | 7 | 2 |
| Sep_14_2017 | 13:10 | hovering | 21 | 7 | 2 |
| Sep_14_2017 | 14:32 | hovering | 21 | 7 | 2 |
| Sep_14_2017 | 16:54 | hovering | 21 | 7 | 2 |

**REFERENCES**

- Adams, D. C., & Otárola-Castillo, E. (2013). Geomorph: An R package for the collection and analysis of geometric morphometric shape data. *Methods in Ecology and Evolution*, 4, 393–399.
- Boehm, M. M. A. (2021). curvr: An R package for measuring total curvature from landmarked specimens. *R package version 0.0.1*. https://github.com/mannfred/curvr
- Rohlf, F. J. (2015). The tps series of software. *Hystrix*, 26.
